# Timing of the fourth dose of RTS,S/AS01 malaria vaccine in perennial settings: a modelling study

**DOI:** 10.1186/s12936-026-05848-6

**Published:** 2026-03-13

**Authors:** Daniella Figueroa-Downing, Sherrie L. Kelly, Aurélien Cavelan, Melissa A. Penny, Josephine Malinga

**Affiliations:** 1 Swiss Tropical and Public Health Institute, Allschwil, Switzerland; 2 University of Basel, Basel, Switzerland; 3 The Kids Research Institute Australia, Nedlands, WA Australia; 4 Centre for Child Health Research, University of Western Australia, Crawley, WA Australia

**Keywords:** Malaria vaccine, Malaria, RTS,S/AS01, Mathematical modelling, Vaccine impact

## Abstract

**Background:**

The recommendation of the RTS,S/AS01 malaria vaccine for use in children in moderate to high transmission areas by the WHO in 2021 provided another crucial tool in reducing malaria morbidity and mortality. As countries introduce the RTS,S/AS01 vaccine, there is an interest in exploring vaccination schedules that align with existing routine health touchpoints and to optimize vaccine coverage.

**Methods:**

This study used OpenMalaria, an individual-based, stochastic model of malaria transmission and disease progression, to assess the impact of different vaccination timing schedules and coverage on uncomplicated malaria cases, severe malaria cases, and malaria-related deaths across different archetypal transmission settings. We ran simulations comparing fourth dose protection against infection and timings at 6-, 9-, 12-, 15- and 18-month intervals following the primary series, including exploring ranges of plausible protection assumptions for many of the dose timings.

**Results:**

The primary series substantially reduces the malaria burden across transmission settings, regardless of timing of the fourth vaccine dose. For example, at a baseline *Plasmodium falciparum* prevalence in 2–10 year olds (*Pf*PR_2-10_) of 20%, our modeling suggests that the primary series is responsible for around 73–93% of the total uncomplicated and 74%-92% of severe cases averted regardless of fourth dose timing in perennial settings. Additionally, a fourth dose of the vaccine in this transmission setting could avert additional 8–38% of uncomplicated and 9–36% of severe malaria cases in addition to the primary series alone. While the number of cases averted varies by baseline prevalence, we find potential flexibility in timing this dose, especially when focused on 6 to 12 months following the primary series.

**Conclusions:**

A fourth dose delivered between 15- and 21-months of age (corresponding to a 6–12 month interval after the third dose) with high population coverage will likely avert the largest proportion of cases of uncomplicated malaria, severe malaria, and deaths in perennial settings, across transmission intensities. Therefore, the delivery of the fourth dose of the RTS,S/AS01 vaccine can be tailored to country-specific context and linked to existing health touchpoints to ensure adequate coverage of this dose to optimize its impact.

**Supplementary Information:**

The online version contains supplementary material available at 10.1186/s12936-026-05848-6.

## Background

Vaccines are one of the most cost-effective public health interventions for reducing morbidity and mortality in children under five, especially in low- and middle- income countries (LMICs) [1]. Since its launch in 1974, vaccination through the Expanded Programme on Immunization (EPI) has helped avert an estimated 146 million deaths in children under 5 years old due to vaccine-preventable diseases, accounting for around 52% of the decline in infant mortality across the African region [2]. Notably, this estimate does not include the impact of the two malaria vaccines—RTS,S/AS01 and R21/Matrix-M—recommended in 2021 and 2023, respectively, for use in children in moderate and high transmission settings [3, 4]. The effectiveness of immunization as a public health tool, depends not only on the availability of efficacious vaccines but also on the successful and timely delivery of all required doses. Optimizing the balance between immunological efficacy, appropriate dosing intervals, and vaccine coverage is therefore critical to achieving the full impact of malaria vaccination programs.

RTS,S/AS01 is a pre-erythrocytic vaccine, a partially protective, infection blocking vaccine that induces an immune response against the circumsporozoite protein of the *Plasmodium falciparum* parasite [5–7]. In Phase 3 clinical trials, RTS,S/AS01 was administered in a 4-dose schedule to 5–17 month olds with the second, third and fourth doses delivered two, three and 20 months after the first dose, respectively [8, 9]. The trials that concluded in 2014 showed a vaccine efficacy against clinical malaria of 36.3% (95% CI 31.8–40.5) and efficacy against severe malaria of 32.2% (95% CI 13.7–46.9) in children 5–17 months old over 48 months of follow up [8]. However, there was, and still is, uncertainty around duration of vaccine protection, ideal deployment schedules for maximum health impact, feasibility of reaching the targeted children and population-level health impact [10, 11].

Building on the Phase 3 clinical trial data, a 2015 multi-model assessment of projected public health impact and cost effectiveness estimated that a four-dose schedule of RTS,S/AS01, deployed in settings with a *Plasmodium falciparum* prevalence in 2–10 year olds (*Pf*PR_2-10_) between 10–65% would avert a median of 116,480 (range 31,450–160,410) clinical cases and 484 (range 189–859) deaths per 100,000 fully vaccinated children. At US$10 per dose, close to the current market price of 9.30 euros [12], a four-dose schedule was predicted to cost US$51 (range $28–437) per clinical case averted and $154 (range $99–487) per death averted [13]. These models assumed vaccine deployment at 5, 7, 9 and 27 months of age, corresponding to an 18-month interval between doses three and four [13]. This led to the 2016 recommendation by the World Health Organization for pilot implementation studies in countries to address remaining questions around feasibility and impact prior to wide-scale introduction.

The recently concluded Malaria Vaccine Implementation Project (MVIP) pilots in Ghana, Kenya and Malawi have provided critical evidence on the implementation realities of introducing a new vaccination schedule into the existing EPI schedule [14–19]. The three countries chose deployment schedules that aligned to their individual country contexts with the primary series delivered between 5 and 9 months of age, and a fourth dose at either 22 or 24 months of age. Over the course of the pilots, coverage of the primary series reached between 60 and 70% of all eligible children in the three sites; however, fourth dose reach was only around 40% in all three original pilots [15, 20]. Despite this, all three countries demonstrated, in a real-world pilot rollout, a 13% reduction in all-cause mortality [8] following vaccine introduction. As early evidence around the implementation challenges of introducing a novel malaria vaccine are surfaced [15, 18, 19, 21, 22], there is a critical gap that additional modeling can fill to help guide global decision making on when and how to best deploy vaccine doses, and particularly a fourth dose of malaria vaccine.

As countries in sub-Saharan Africa continue to introduce one of the two licensed malaria vaccines, there is a continued interest in exploring dose schedules aligned to existing EPI touchpoints to optimize vaccination coverage. For example, Ghana ultimately shifted their fourth dose to children 18-months of age to be given alongside the second dose of the measles vaccine after early pilot programs showed high dropout for a fourth dose delivered beyond the existing EPI schedule [16]. After this change, the coverage of the fourth vaccine dose in Ghana rose from 42% [20] to 81% as of 2022 [23].

In this study, we provide model-based evidence for decisions around timing and deployment of a fourth dose of RTS,S/AS01 in perennial settings, using age-based vaccination strategies. We use mathematical modeling to estimate impact of a range of timings of the fourth dose of RTS,S/AS01 vaccine on malaria morbidity and mortality in children under five years of age in settings with varying transmission intensity. We use modeling to outline considerations for maximizing the health impact of timing of the fourth dose based on both coverage and immunological response, for different malaria-endemic settings.

## Methods

### Transmission model

We used OpenMalaria (https://github.com/OpenMalaria-Org/openmalaria), an individual-based, stochastic model of malaria epidemiology and control. The model simulates mosquito dynamics, vector infectiousness, dynamics of infection to humans, and at the individual level models blood-stage parasite densities and immunity dynamics The model includes a pathogenesis module that allows estimation of the incidence of malaria morbidity including severe disease and hospitalizations, and mortality [24, 25]. The model was originally developed to evaluate the population-level health impact of the RTS,S/AS01 malaria vaccine [13, 26]. In OpenMalaria, we model the deployment of a pre-erythrocytic vaccine like RTS,S/AS01 as the prevention of sporozoites and therefore, infection. Additional model components and assumptions are detailed in Supplemental Table S1. The model was calibrated to clinical incidence of malaria from the RTS,S/AS01 Phase 3 clinical trial to derive estimates of initial protection against infection after the three-dose primary series, duration of protection (half-life, or the time it takes the initial protection against infection to reduce by 50%), and subsequent protection provided by a fourth dose [11].

### Settings and simulation scenarios

To align with previous modeling used to support WHO malaria vaccine policy, including the 2016 WHO Position Paper on Malaria Vaccines [27], we simulated archetypal transmission settings to provide the full range of probable scenarios and outcomes represented across sub-Saharan Africa [13]. A factorial design was used to assess the impact of a fourth dose of RTS,S/AS01 in an archetypal perennial setting. Simulated parameters include varying malaria transmission intensity (*Pf*PR_2–10_: 10%–60%, access to treatment for symptomatic cases (45%, 70%), and immunization coverage, comprising around 300 scenarios, which are described in Table 1. Simulations were run in a population of 50,000 individuals, assuming constant population growth and demography, no scale-up of vaccination coverage over time, and year-on-year immunization rates were held constant. We assumed no change in the level of other interventions during the immunization implementation period. We report model results for a range of transmission intensities, namely *Pf*PR_2–10_ ranging between 10 and 60%.

**Table 1:** Summary of simulations: variables and levels

Variable	Parameter description		Parameter value
Time horizon	Total intervention period		10 years
EIRs	Range of EIRs corresponding to *Pf*PR in 2–10 year olds of:		10%, 20%, 30%, 40%, 50%, 60%
Access to uncomplicated case management^a^	Default access		45%
	High access		70%
Vaccine deployment scenarios	Third dose (representing primary series)		9 months of age
	Fourth dose at 6-month interval		15 months of age
	Fourth dose at 9-month interval		18 months of age
	Fourth dose at 12-month interval		21 months of age
	Fourth dose at 15-month interval		24 months of age
	Fourth dose at 18-month interval		27 months of age
Vaccine properties	Protection against infection^b^:	Dose 3 (primary series):	91.1% [95% CI 74.5–99.7%] [11]
		Dose 4, 6-month interval:	Upper bound: 91.1% [95% CI 74.5–99.7%] Lower bound: 77% [95% CI 68.3–86.6%]
		Dose 4, 9-months interval:	Upper bound: 91% [95% CI 74.5–99.7%] Lower bound: 77% [95% CI 68.3–86.6%]
		Dose 4, 12-month interval:	77% [95% CI 68.3–86.6%] [33]
		Dose 4, 15-month interval:	Upper bound: 77% [95% CI 68.3–86.6%] Lower bound: 49% [95% CI 32–68.6%]
		Dose 4, 18-month interval:	49% [95% CI 32–68.6%] [11]
	Half-life (months), all		7.32 [95% CI 6–10 months] [11]
	Weibull decay shape parameter		k = 0.69 (bi-phasic, quick decay followed by slow decay) [11]
Vaccination coverage scenarios	High coverage scenario *(assuming no drop out)*		Dose 3 coverage: 80% Dose 4 coverage 80%
	High coverage and high dropout scenario *(assuming 50% drop out)*		Dose 3 coverage: 80% Dose 4 coverage: 40%
	Low coverage scenario *(assuming no drop out)*		Dose 3 coverage: 50% Dose 4 coverage 50%

^a^ Probability of access to treatment for uncomplicated disease during a 14-day period

^b^ The represents the absolute protection against infection achieved by the additional dose and not a percentage of third dose

### Vaccine deployment scenarios

We modeled a three-dose, age-based primary series of RTS,S/AS01 with the third dose delivered at 9 months of age, in line with immunization schedules used during the MVIP pilots [20] and earlier studies [11]. A fourth age-based dose was evaluated at multiple time points in the second year of life: specifically at 15, 18, 21, 24, and 27 months, representing intervals of 6 to 18 months after the primary series. These time points were selected based on stakeholder consultations and aligned with existing routine childhood vaccination visits in the first three years of life. These dose timings correspond to select countries delivering meningitis A vaccine at 15 or 18 months old [28]; second dose of measles vaccine at 18 months old [29]; 21 months of age to roughly reproduce Malawi’s schedule in the MVIP pilots (in Malawi the fourth dose was given at 22 months of age) [20, 30]; Kenya’s schedule in the MVIP pilot at 24 months [20], and at 27 months to reflect the original modeled impact from the Phase 3 clinical trials [13]. We did not consider seasonal settings where additional doses (five or more doses) are recommended [3]; however the force of infection is higher in the peak seasonal months so the dose timing and numbers would need to be optimally tailored to those epidemiological patterns [31].

### Vaccine coverage and protection against infection

To examine potential differences in the impact of dose timing by vaccine coverage, we simulated three coverage scenarios: (a) a high coverage scenario with the primary series coverage of 80% and no dropout between the primary series and fourth dose (four-dose coverage of 80%); (b) a high coverage but high dropout scenario with the primary series coverage of 80% and a 50% dropout between the primary series and fourth dose (four-dose coverage of 40%, or roughly what was measured during early MVIP pilots); (c) a low coverage scenario with the primary series coverage of 50% and no dropout between the primary series (four-dose coverage of 50%).

We assumed that protection against infection is at its maximum following the third dose of the primary series based on the Phase 3 trial data [8] and aligned with previous modeling work using OpenMalaria and other malaria vaccine models [11, 13, 32]. This was estimated at 91% based on calibration of our model to the RTS,S/AS01 Phase 3 clinical trial data (Table 1) [11]. We assumed protection decays over time following a biphasic pattern (shape parameter k = 0.69), representing rapid initial waning followed by a slower long-term decay [11]. The same decay pattern is assumed for the fourth dose, regardless of dose timing.

Finally, for the fourth dose, we based protection against infection assumptions on existing data, and logical bounds. The protection against infection for a fourth dose delivered at an 18-month interval after the primary series was based on the calibration to Phase 3 clinical trial data by Penny et al. [11]. The protection against infection of a fourth dose delivered at a 12-month interval after the primary series is based on recent work on calibrating clinical trial data to OpenMalaria by Malinga et al. [33] from recent field trials delivering RTS,S/AS01 with and without SMC [10, 34]. Without existing data on efficacy assumptions for doses delivered at 6-, 9-, and 15-month intervals, we explored the impact of both low and high protection against infection scenarios. This range was defined by the upper and lower protection estimates of surrounding dose timings, based on previous modeling [11, 33].

### Outcome measures

Public health impact was calculated as cumulative events averted per 100,000 fully vaccinated children, and age-based incidence for each outcome over a 10-year time period. We examined the impact of each of the fourth dose timings on uncomplicated cases, severe cases, direct malaria deaths, and all malaria deaths (including those where malaria infection contributes to the outcome, in the presence of other co-occurring conditions [5, 20]). Results were presented by age and across a range of *Pf*PR_2-10_, for children under 5 years of age. ‘Fully vaccinated children’ is defined as the number of children receiving at least the three-dose primary series of RTS,S/AS01 vaccine. The proportion of additional cases averted by dose strategy was calculated as the proportion of cases averted by the fourth dose compared to cases averted by the primary series alone under each given scenario. All scenarios were compared against a baseline counterfactual with no malaria vaccine intervention. Impact estimates were computed as median estimates across 10 stochastic realizations. Where we report a range of expected impact for *Pf*PR_2-10_ of 10%, 20% and 30%, these numbers represent the range of median impact across all fourth dose timings and plausible protection against infection values, unless otherwise noted. Results for all modeled transmission levels *Pf*PR_2-10_ of 10%-60%, are shown in the Figures and the Supplement.

## Results

**Fig. 1:**
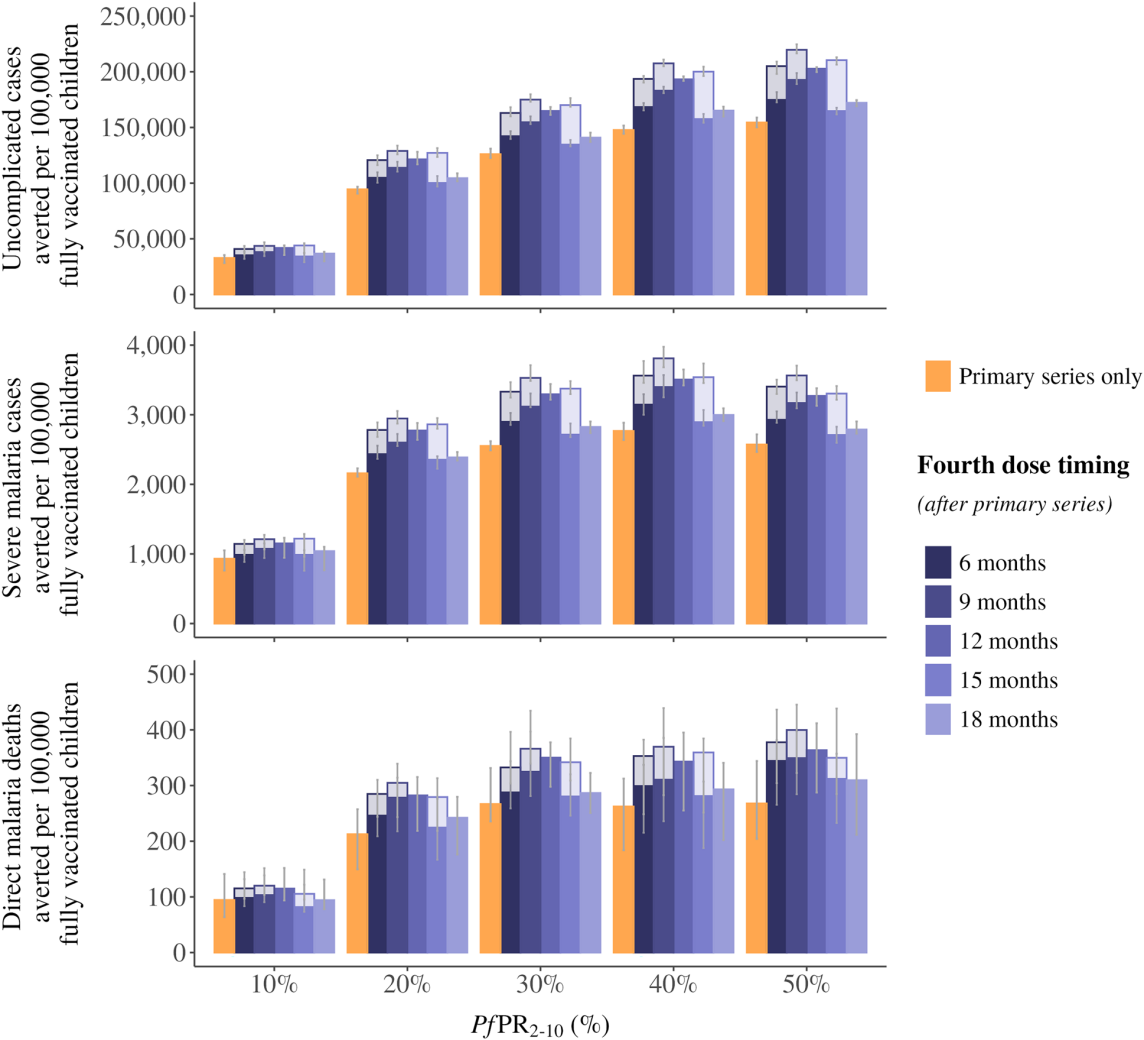
Events averted per 100,000 fully vaccinated children by RTS,S/AS01 malaria vaccine dose timing. The median uncomplicated malaria cases averted (**A**), severe malaria cases averted (**B**), and direct malaria deaths averted (**C**) in children under 5 years of age per 100,000 fully vaccinated children comparing the three-dose primary series and a fourth dose delivered at a 6-, 9-, 12-, 15- or 18-month interval after the primary series. The two shades on the 6-, 9-, and 15-month intervals represent the range of probable protection against infection values for these three dose timings. Cases averted are calculated over a 10-year time horizon. This is a default reference scenario of high coverage, where primary series coverage is assumed at 80% of the target population and there is no drop-out between the primary series and the fourth dose. Error bars show 95% confidence intervals across stochastic replicates. Access to care is represented as a 45% 14-day probability to receive treatment for uncomplicated malaria

The three-dose primary series substantially reduced the malaria burden across all transmission intensities, regardless of timing of the fourth dose (Fig. 1). In the high coverage scenario, with primary series coverage of 80% and no dropout between the third and fourth dose, the primary series accounted for 76–95% of total uncomplicated cases averted in a setting with baseline *Pf*PR_2-10_ of 10%, 73–93% of uncomplicated cases in *Pf*PR_2-10_ of 20% and 72–93% of total uncomplicated cases in *Pf*PR_2-10_ of 30%. Very similarly, the three-dose primary series accounted for 76–95%, 74–92%, and 72–93% of severe malaria cases averted at *Pf*PR_2-10_ of 10%, 20% and 30%, respectively.

**Fig. 2:**
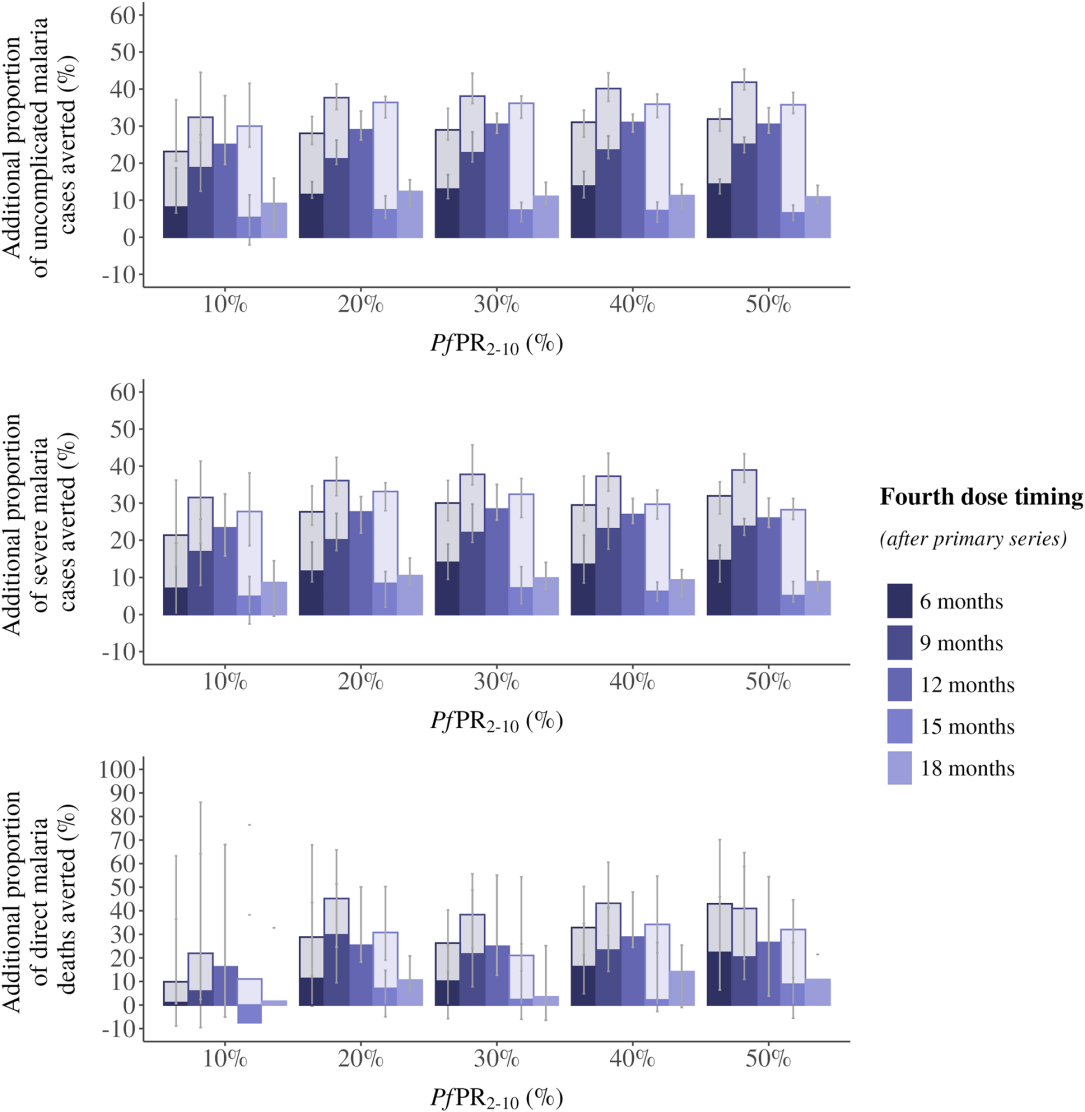
Proportion of additional cases averted by a fourth dose of RTS,S/AS01 malaria vaccine. The median proportion of additional uncomplicated malaria cases averted (**A**), severe malaria cases averted (**B**), and direct malaria deaths averted (**C**) in children under 5 years of age per 100,000 fully vaccinated children at different timings of a fourth dose delivered at a 6-, 9-, 12-, 15- or 18-month interval after the primary series, compared to the primary series alone. The two shades on the 6-, 9-, and 15-month intervals represent the range of probable protection against infection values for these three dose timings. Cases averted are calculated over a 10-year time horizon. This is a default reference scenario of high coverage, where primary series coverage is assumed at 80% of the target population and there is no drop-out between the primary series and the fourth dose. Error bars show 95% confidence intervals across stochastic replicates. Access to care is represented as a 45% 14-day probability to receive treatment for uncomplicated malaria.

While the primary series is responsible for the majority of the impact in an age-based deployment of RTS,S/AS01, our modeling shows that a deployment of a fourth dose, especially between 6 and 12 months after the primary series, would likely contribute to a substantial number of additional uncomplicated and severe cases and deaths averted (Fig. 2). Across all transmission settings, a fourth dose delivered 6, 9 or 12 months after the primary series provide the greatest impact, though the absolute benefit varies depending on the underlying protection against infection of the dose. As we do not have empirical evidence on how the fourth dose efficacy is impacted by dose spacing, we present a plausible range of efficacies that suggest that the impact of any dose in the 6 to 12 month interval would likely be similar.

In moderate to high transmission intensities, we find that a fourth dose delivered between 6 and 18 months could avert additional cases. In settings with a *Pf*PR_2-10_ of 10%, we estimate an additional 6–32% of uncomplicated cases averted compared to primary series alone, at a *Pf*PR_2-10_ of 20% an additional 8–38% of uncomplicated cases averted and at a *Pf*PR_2-10_ of 30% an additional 8-39% averted. We estimate 5–32%, 9–36%, and 7–38% more severe cases averted by the fourth dose compared to the primary series alone, across dose timing at *Pf*PR_2-10_ of 10%, 20% and 30%, respectively (Fig. 2). These large ranges are driven by the combination of different fourth dose timings and the range of assumptions around initial vaccine protection against infection.

However, when we consider a single dose timing in the optimal range, such as a fourth dose delivered 12 months after the primary series, we estimate that dose timing would avert roughly one-quarter to one-third more events than the primary series alone—25%–31% more uncomplicated cases, 23%–28% more severe cases, and 16%–29% more deaths—in settings with *Pf*PR_2-10_ of 10% to 50%. In comparison, a fourth dose delivered 18 months after the primary series would likely avert an additional 9%–12% of uncomplicated cases and 9%–11% of severe cases in the same transmission settings.

Our findings show there is an interaction between dose spacing and the assumptions of the underlying protection against infection that drives value uncertainty in our estimates. We present modeling results for differing protection assumptions with plausible ranges of the fourth dose given at 6-, 9-, 12- and 15- months following the primary series and investigate the public health impact. While the impact remains high and is similar if the fourth dose is given 6 to 12 months after the primary doses, we find different conclusions given a single ideal dose timing. For instance, the 9-month interval (corresponding to 18-months of age at vaccination) could provide the highest cumulative impact as protection is extended to the second year or could be less impactful than a 12-month interval, depending on protection against infection assumptions.

Critically, there is even greater uncertainty around the impact of a 15-month interval (a fourth dose given at 24-months of age). The potential impact of this dose timing is highly dependent on its corresponding level of protection against infection (Figs. 1 and 2). Specifically, in this study, if the 15-month interval provides the same protection against infection as a 12-month interval then, all else being equal, the slightly longer interval of 15- versus 12-months would lead to a higher cumulative impact over time as a later dose would maintain higher level of protection into the third year of life. However, if the protection against infection is closer to what was observed at the 18-month interval then a fourth dose at the 15-months interval would provide the lowest cumulative impact in most transmission settings. This makes sense considering the trade-off between protection against infection, duration of protection, and the underlying patterns of disease driven by ongoing exposure to *P. falciparum*. A more accurate estimate of impact at this time interval would require further real-world data to better inform the vaccine protection and hence the modeled impact estimates.

**Fig. 3:**
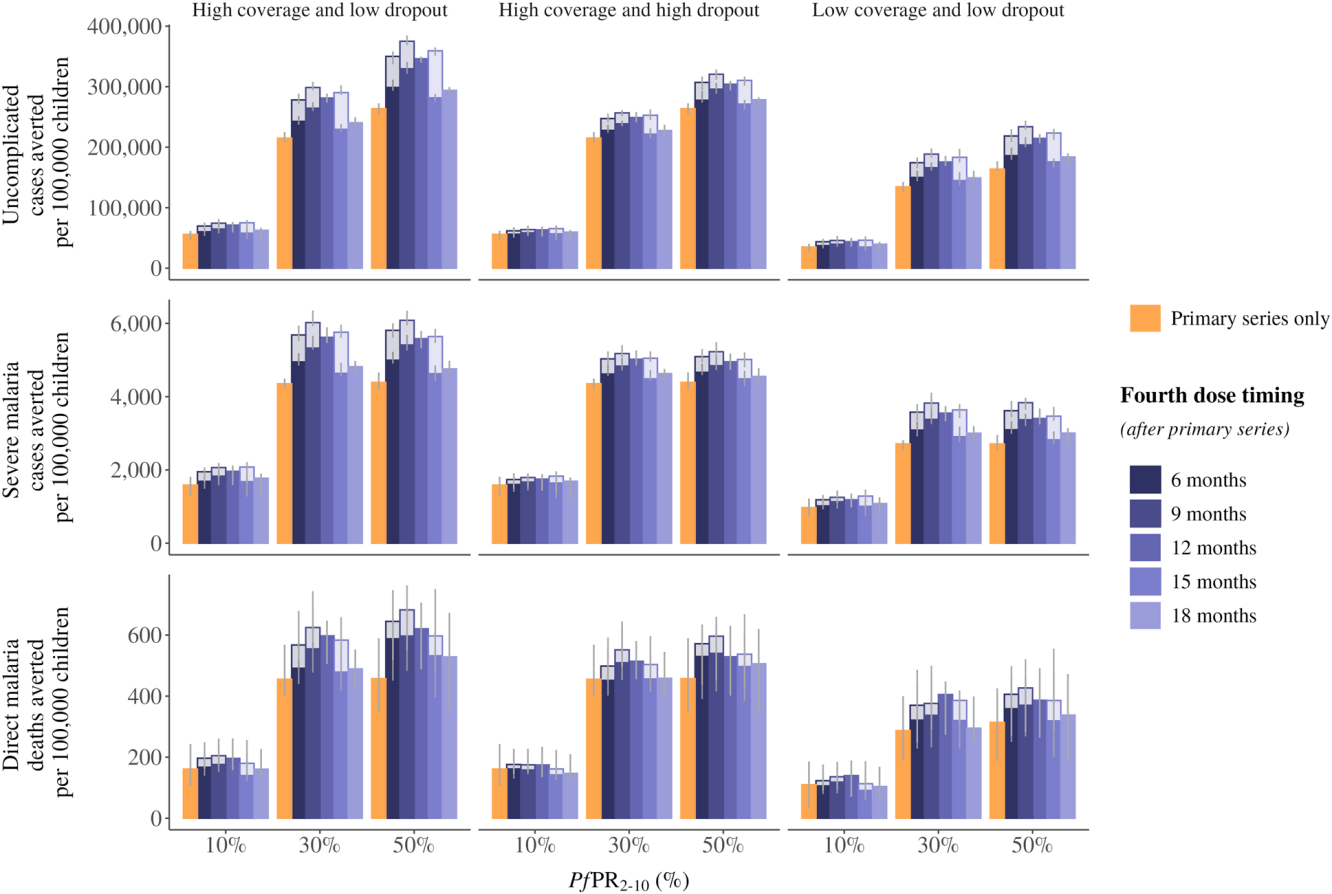
Events averted per 100,000 children under different vaccine coverage rates. The median uncomplicated malaria cases averted (**A**), severe malaria cases averted (**B**), and direct malaria deaths (**C**) averted in children under 5 years of age per 100,000 children comparing the three-dose primary series and a fourth dose delivered at a 6-, 9-, 12-, 15- or 18-month interval after the primary series. Results are shown for three coverage scenarios: high coverage (left) with the primary series coverage of 80% and no dropout between the primary series and fourth dose (fourth dose coverage of 80%); high coverage but high dropout (centre) with the primary series coverage of 80% and a 50% dropout between the primary series and fourth dose (fourth dose coverage of 40%) and; low coverage (right) with the primary series coverage of 50% and no dropout between the primary series (fourth dose coverage of 50%). The two shades on the 6-, 9-, and 15-month intervals represent the range of probable protection against infection values for these three dose timings. Cases averted are calculated over a 10-year time horizon. Error bars show 95% confidence intervals across stochastic replicates. Access to care is represented as a 45% 14-day probability to receive treatment for uncomplicated malaria

The proportion of children reached with both the primary series and the fourth dose, measured as vaccine coverage, is a crucial driver for impact against malaria morbidity and death. The overall magnitude of the impact, regardless of transmission intensities, is substantially reduced when the reach of the primary series is not sufficiently high and moderately reduced when the fourth dose reach is limited in the case of a high dropout scenario (Fig. 3). We estimate that in a moderate transmission intensity with *Pf*PR_2-10_ of 20%, a third-dose coverage of 50% instead of 80% would result in around 37% reduction in impact on both uncomplicated and severe malaria cases, while a coverage drop out of 50% from primary series to fourth dose when delivered at a 12-month interval, even with a high third dose reach, would result in around 10% reduction in impact on both uncomplicated and severe malaria. In an alternate scenario where we assume the paediatric population has much higher probability of receiving timely treatment for uncomplicated malaria at 70%, we found that sufficient reach with the primary series remains the most important driver of impact regardless of transmission setting **(**Supplemental Figure S8).

**Fig. 4:**
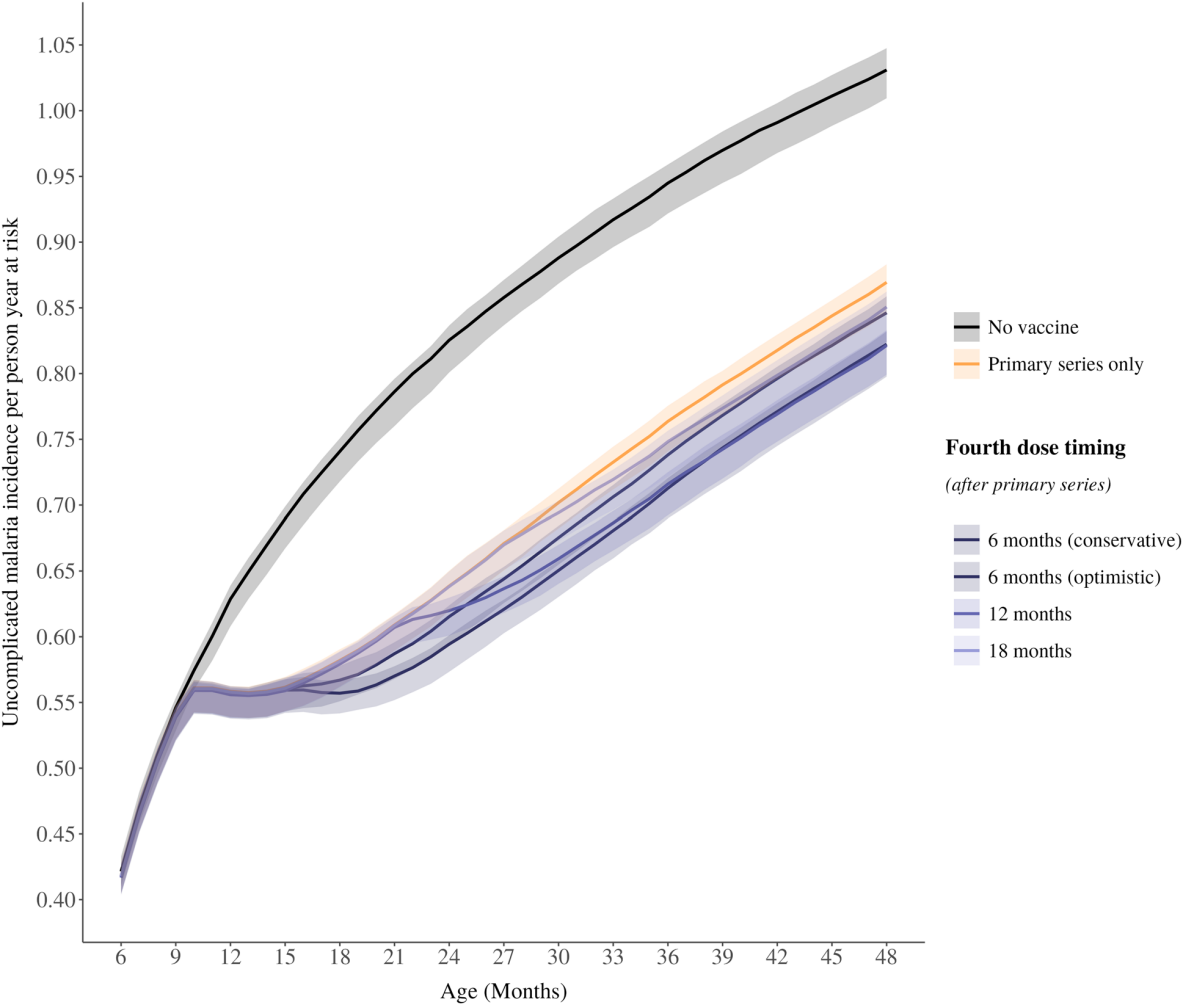
Cumulative incidence rates of uncomplicated malaria cases by age in months, PfPR_2-10_ = 20%. The cumulative incidence rate of uncomplicated malaria cases averted by age in months, over 10 years of follow up. The results show the total effects on the age-patterns of uncomplicated disease for a no intervention scenario (black line), three-dose primary series (yellow line), and a fourth dose delivered at a 6-, 12- or 18-month interval after the primary series (purple lines). The shaded areas show 95% confidence intervals across stochastic replicates. Results are shown for a single baseline transmission level of PfPR_2-10_ of 20%, with access to care represented as a 45% 14-day probability to receive treatment for uncomplicated malaria

Finally, it is important to highlight the children who remain unprotected due to being age ineligible (i.e. too young to be vaccinated) in whom a substantial burden of malaria disease and death still remains. The age-specific impact is largely concentrated between 9 and 30 months of age (Fig. 4 and Supplemental Figures S6-7), with the timing of impact differing by dose timings. Since the burden of symptomatic uncomplicated cases, severe cases and deaths are concentrated in younger children, it stands to reason that a fourth dose of RTS,S/AS01 vaccine targeting this high-burden age range will have the greatest impact.

## Discussion

Our modeling results show that RTS,S/AS01 malaria vaccine health impact largely depends on the number of children reached with the vaccine for both the three-dose primary series and the fourth dose. To maximize lives saved, achieving high coverage of the primary series should remain a top priority, alongside efforts to ensure the fourth dose is accessed. Regardless of vaccine coverage, our analysis confirm that a fourth dose deployed between 6 and 12 months after the primary series leads to the highest burden averted. However, both the absolute and relative magnitude of impact of the fourth dose is maximized when more targeted children are vaccinated with the fourth dose. Hence, the fourth dose should be timed to prioritize touchpoints that will maximize coverage. While the absolute magnitude of impact may vary, these conclusions remain across transmission intensities. Overall, our results support the flexible timing of a fourth dose of RTS,S/AS01 malaria vaccine to align with an existing immunization touchpoint in the second year of life to maximize coverage and optimize the impact in perennial settings.

A recent modeling study by Thompson et al. looking at dose timing in seasonal settings came to a similar conclusion around potential flexibility in dose timing, with the aim of improving access to the vaccine in seasonal settings [31]. These conclusions align with the understanding of the age-distribution of severe disease and death, where the burden remains highly concentrated in young children under two years of age [35, 36]. Critically, the current vaccination schedule for malaria vaccines continues to leave an important portion of children unprotected due to being too young for the current vaccine (i.e. less than five or six months old at the start of the primary series) (Fig. 4).

Our results for the absolute burden averted as shown for the different coverage scenarios reinforce the crucial importance of the proportion of children reached with the vaccine. While we estimate different magnitudes of impact based on incrementally different dose timings and plausible protection against infection, we did not find any scenario where a high vaccine protection against infection and optimized dose timing under low coverage assumptions outperformed a higher coverage scenario. Ultimately, reaching a sufficiently large number of children with the RTS,S/AS01 vaccine by leveraging existing systems should be prioritized to achieve desired impact. This will, hopefully, allow for flexibility in the timing of the fourth dose to align to country-specific schedules and needs.

The protection against infection for 6-, 9- and 15- month intervals are presented as a plausible range and for the simplicity of the simulations, we only simulated the extremes of the plausible ranges based on existing evidence. Based on immunological data from the Phase 3 trial [8] and previous calibration [11] studies have shown that the fourth dose provides some return to protection; however, we do not know to what level and how both the fourth dose interval after the third as well as the age of vaccination effect this “boosting” effect. With this approach, we attempt to demonstrate the trade-off between dose timing against underlying age patterns of disease and immunological protection.

This trade-off is especially highlighted by the different conclusions that can be drawn across the wide range of potential protection against infection for the 15-month dosing interval. This interval corresponds to a dose given at 24-months of age, which is of high interest to many countries as it could contribute to strengthening a second-year of life platform alongside other booster and catch-up vaccines [37]. Unfortunately, the bounds on the assumptions of protection against infection are high due to lack of data to inform vaccine assumptions, and therefore, this dose could potentially provide the most or the least cumulative impact depending on the true protection against infection of this dose timing. Because of the wide plausible efficacy range at this interval, countries considering a fourth dose at 24-months of age may benefit most from additional real-world evidence on this dose timing.

While earlier fourth dose vaccination would lead to higher absolute impact, our modeling study also shows important implications for catch-up or delayed vaccination. While we did not explicitly model a catch-up campaign delivered at a later time point, our estimates show that a fourth dose delivered up to 27 months of age would still avert additional cases beyond the primary series alone. Aiming to reach children 6–12 months following the primary series would still be the preferred operational approach; however, if a child is not reached in this timeframe, then a program can still consider reaching them in the third year of life as still potentially impactful.

As with all modeling studies, there are several limitations in our study including necessary simplification of modeling assumptions and insufficient data to inform protection assumptions of vaccine timings outside existing evidence. Firstly, our study aimed to provide additional evidence to inform global policy setting by exploring multiple scenarios across a range of malaria transmissions and levels of access to case management for symptomatic cases to identify any important trends or signals. Therefore, we model a set of archetypal perennial settings and assume a fixed level of access to care, monthly and annual transmission, and population-level access to the vaccine. This modeling is not meant to recreate specific country scenarios, which would be best carried out by or in close collaboration with the individual country; instead, it sets to define the broad bounds within which a global policy decision can be taken.

Secondly, our model estimates rely on assumptions around the initial protection against infection at different dose timings. However, data to inform these parameters are only available for the primary series [11, 13], 12-month interval [33], and 18-month interval [11, 13]. These results are aligned with results from other models regarding original calibration to the Phase 3 trials [13], and updated calibration of models to newer real-world implementation data [31]. However, there is no evidence-based estimates for vaccine protection against infection at intervals shorter than 12-months post-primary series, nor for the 15-month interval. To mitigate the limitations around the protection against infection assumption, we present a range of plausible values around these implementation scenarios to explore the possible public health impact. Still, based on our model structure and the available evidence, we are unable to assume any differences in immunological response for shorter time intervals, which could affect our impact assessments. These estimates should be updated once additional evidence on alternate dose timings become available.

Thirdly, we use OpenMalaria, an established and validated individual-based model of malaria transmission and intervention dynamics for this study. However, as with all models there are structural limitations. Relevant to this study are model parameters that determine age-patterns of severe disease and deaths that were calibrated to older data of severe disease, while we know patterns of underlying disease and co-morbidities may have changed over time. Nevertheless, a recent comparison of our model with contemporary data on severe disease from Kenya Health and Demographic Surveillance Sites (HDSS) [38] shows that the model is still able to recover severe malaria trends seen in contemporary real-world data.

Taking these results in context, countries are increasingly considering use of malaria vaccines alongside perennial malaria chemoprevention (PMC) in children which was outside of the scope of this. PMC (formerly IPTi), is conditionally recommended by WHO for use in children 3–24 months of age in moderate to high transmission perennial settings [39], although modeling has suggested that expanding that age range could increase impact [40]. Other modeling studies have estimated the potential effects of combined RTS,S/AS01 and PMC [41], but we did not include this combination in this study. There was no policy recommendation on combining malaria vaccines and PMC at the time of this analysis and data on feasibility, efficacy and safety of co-administration is ongoing [42]. Once these data are available, further analysis would be able to inform our modeling so we can estimate combined impact to fill evidence gaps identified by the global community [43].

These considerations of timing of additional doses of another vaccine, or injectable, are relevant beyond the RTS,S/AS01 context. There are other pre-erythrocytic vaccines in Phase 2 and Phase 3 trials, as well as a steady pipeline of forthcoming vaccines and injectable products [44–46]. As the second licensed malaria vaccine, R21/Matrix-M is also introduced across the sub-continent, similar questions have already arisen around optimal deployment of the vaccine. While modeling based on immune correlates has shown a different protection profile than RTS,S/AS01 vaccine [47], expanding the reach of this vaccine will likely remain the most important determinant of population-level health impact. The effective and impactful deployment of any new intervention will depend on finding the delicate balance between optimized immunological benefit and realistic delivery strategies, to maximize real world impact.

## Conclusions

To maximize the public health impact of the RTS,S/AS01 malaria vaccine, countries should prioritize achieving high coverage of the three-dose primary series, followed by a flexibly timed fourth dose to ensure higher coverage of this fourth dose. Our modeling in perennial settings shows that vaccine impact is primarily driven by the number of children reached, regardless of the exact timing of the fourth dose. When high coverage is achieved, delivering a fourth dose between 6 and 12 months after the third dose consistently results in the greatest burden averted. These findings hold across transmission intensities, reinforcing that the fourth dose should be aligned with existing immunisation touchpoints within the 15–24-month age range to maximize vaccine uptake. If strong touchpoints do not exist, it would be prudent to establish those to increase access to all doses of the malaria vaccine. As real-world rollout of malaria vaccines that include RTS,S/AS01 and R21/Matrix-M expands, evaluating the effectiveness of flexible fourth dose strategies will be key to informing national implementation and optimising long-term malaria control.

## Supplementary Information

Supplementary material 1.

## Data Availability

Model inputs and scripts supporting the conclusions of this article are available at [10.5281/zenodo.17342794] (https:/doi.org/10.5281/zenodo.17342794).

## References

[1] Munk C, Portnoy A, Suharlim C, Clarke-Deelder E, Brenzel L, Resch SC, et al. Systematic review of the costs and effectiveness of interventions to increase infant vaccination coverage in low- and middle-income countries. BMC Health Serv Res. 2019;19(1):741. 31640687 10.1186/s12913-019-4468-4 PMC6806517

[2] Shattock AJ, Johnson HC, Sim SY, Carter A, Lambach P, Hutubessy RCW, et al. Contribution of vaccination to improved survival and health: modelling 50 years of the Expanded Programme on Immunization. Lancet. 2024;403(10441):2307–16. 38705159 10.1016/S0140-6736(24)00850-X PMC11140691

[3] World Health Organization. Malaria vaccine: WHO position paper – March 2022 [Internet]. 2022 [cited 2025 Jul 25] p. 61–80. (Weekly epidemiological record). Report No.: 9. Available from: https://www.who.int/publications/i/item/who-wer9709-61-80

[4] World Health Organization. Malaria vaccine: WHO position paper – May 2024 [Internet]. 2024 [cited 2025 Jul 25] p. 225–48. (Weekly epidemiological record). Report No.: 19. Available from: https://www.who.int/publications/i/item/who-wer-9919-225-248

[5] Casares S, Brumeanu TD, Richie TL. The RTS,S malaria vaccine. Vaccine. 2010;28(31):4880–94. 20553771 10.1016/j.vaccine.2010.05.033

[6] Cohen J, Nussenzweig V, Nussenzweig R, Vekemans J, Leach A. From the circumsporozoite protein to the RTS, S/AS candidate vaccine. Hum Vaccin. 2010;6(1):90–6. 19806009 10.4161/hv.6.1.9677

[7] Laurens MB. RTS,S/AS01 vaccine (Mosquirix™): an overview. Hum Vaccines Immunother. 2020;16(3):480–9. 10.1080/21645515.2019.1669415 PMC7227679 31545128

[8] RTS,S Clinical Trials Partnership. Efficacy and safety of RTS,S/AS01 malaria vaccine with or without a booster dose in infants and children in Africa: final results of a phase 3, individually randomised, controlled trial. Lancet Lond Engl. 2015;386(9988):31–45. 10.1016/S0140-6736(15)60721-8 PMC5626001 25913272

[9] RTS,S Clinical Trials Partnership. Efficacy and safety of the RTS,S/AS01 malaria vaccine during 18 months after vaccination: a phase 3 randomized, controlled trial in children and young infants at 11 African sites. PLoS Med. 2014;11(7):e1001685. 25072396 10.1371/journal.pmed.1001685 PMC4114488

[10] Cairns M, Barry A, Zongo I, Sagara I, Yerbanga SR, Diarra M, et al. The duration of protection against clinical malaria provided by the combination of seasonal RTS,S/AS01E vaccination and seasonal malaria chemoprevention versus either intervention given alone. BMC Med. 2022;20(1):352. 36203149 10.1186/s12916-022-02536-5 PMC9540742

[11] Penny MA, Pemberton-Ross P, Smith TA. The time-course of protection of the RTS,S vaccine against malaria infections and clinical disease. Malar J. 2015;14:437. 26537608 10.1186/s12936-015-0969-8 PMC4634589

[12] UNICEF Supply Division. Malaria Vaccine: Questions and Answers on Vaccine Supply, Price, and Market Shaping Update [Internet]. 2023. Available from: https://www.unicef.org/supply/media/22311/file/Malaria-Vaccine-Market-QA-July-2023.pdf

[13] Penny MA, Verity R, Bever CA, Sauboin C, Galactionova K, Flasche S, et al. Public health impact and cost-effectiveness of the RTS,S/AS01 malaria vaccine: a systematic comparison of predictions from four mathematical models. Lancet. 2016;387(10016):367–75. 26549466 10.1016/S0140-6736(15)00725-4 PMC4723722

[14] Praet N, Asante KP, Bozonnat MC, Akité EJ, Ansah PO, Baril L, et al. Assessing the safety, impact and effectiveness of RTS,S/AS01E malaria vaccine following its introduction in three sub-Saharan African countries: methodological approaches and study set-up. Malar J. 2022;21(1):132. 35468801 10.1186/s12936-022-04144-3 PMC9036501

[15] Asante KP, Mathanga DP, Milligan P, Akech S, Oduro A, Mwapasa V, et al. Feasibility, safety, and impact of the RTS,S/AS01E malaria vaccine when implemented through national immunisation programmes: evaluation of cluster-randomised introduction of the vaccine in Ghana, Kenya, and Malawi. The Lancet. 2024;403(10437):1660–70. 10.1016/S0140-6736(24)00004-7 PMC11718159 38583454

[16] Adjei MR, Amponsa-Achiano K, Okine R, Tweneboah PO, Sally ET, Dadzie JF, et al. Post introduction evaluation of the malaria vaccine implementation programme in Ghana, 2021. BMC Public Health. 2023;23(1):586. 36991394 10.1186/s12889-023-15481-6 PMC10052308

[17] Hill J, Bange T, Hoyt J, Kariuki S, Jalloh MF, Webster J, et al. Integration of the RTS,S/AS01 malaria vaccine into the Essential Programme on Immunisation in western Kenya: a qualitative longitudinal study from the health system perspective. Lancet Glob Health. 2024. 10.1016/S2214-109X(24)00013-5. 38430916 10.1016/S2214-109X(24)00013-5 PMC10932755

[18] Hoyt J, Okello G, Bange T, Kariuki S, Jalloh MF, Webster J, et al. RTS,S/AS01 malaria vaccine pilot implementation in western Kenya: a qualitative longitudinal study to understand immunisation barriers and optimise uptake. BMC Public Health. 2023;23(1):2283. 37980467 10.1186/s12889-023-17194-2 PMC10657022

[19] Grant J, Gyan T, Agbokey F, Webster J, Greenwood B, Asante KP. Challenges and lessons learned during the planning and early implementation of the RTS,S/AS01E malaria vaccine in three regions of Ghana: a qualitative study. Malar J. 2022;21(1):147. 35550113 10.1186/s12936-022-04168-9 PMC9096766

[20] Malaria Vaccine Implementation Programme (MVIP) Programme Advisory Group (PAG). Full Evidence Report on the RTS,S/AS01 Malaria Vaccine. SAGE Yellow Book for October 2021: World Health Organization; 2021

[21] Okyere J, Bediako VB, Ackah JA, Acheampong E, Owusu BA, Agbemavi W, et al. RTS,S/AS01E vaccine defaults in Ghana: a qualitative exploration of the perspectives of defaulters and frontline health service providers. Malar J. 2023;22(1):260. 37674197 10.1186/s12936-023-04690-4 PMC10483715

[22] Simbeye AJ, Kumwenda S, Cohee LM, Omondi D, Masibo PK, Wao H, et al. Factors associated with malaria vaccine uptake in Nsanje district, Malawi. Malar J. 2024;23(1):105. 38627704 10.1186/s12936-024-04938-7 PMC11022426

[23] Osoro CB, Ochodo E, Kwambai TK, Otieno JA, Were L, Sagam CK, et al. Policy uptake and implementation of the RTS,S/AS01 malaria vaccine in sub-Saharan African countries: status 2 years following the WHO recommendation. BMJ Glob Health. 2024;9(4):e014719. 38688566 10.1136/bmjgh-2023-014719 PMC11085798

[24] Smith T, Killeen GF, Maire N, Ross A, Molineaux L, Tediosi F, et al. Mathematical modeling of the impact of malaria vaccines on the clinical epidemiology and natural history of *Plasmodium falciparum* malaria: overview. Am J Trop Med Hyg. 2006;75(2_suppl):1–10. 16931810 10.4269/ajtmh.2006.75.2_suppl.0750001

[25] Ross A, Maire N, Molineaux L, Smith T. An epidemiologic model of severe morbidity and mortality caused by *Plasmodium falciparum*. Am J Trop Med Hyg. 2006;75(2 Suppl):63–73. 16931817 10.4269/ajtmh.2006.75.63

[26] Reiker T, Golumbeanu M, Shattock A, Burgert L, Smith TA, Filippi S, et al. Emulator-based Bayesian optimization for efficient multi-objective calibration of an individual-based model of malaria. Nat Commun. 2021;12(1):7212. 34893600 10.1038/s41467-021-27486-z PMC8664949

[27] Malaria vaccine: WHO position paper-January 2016 - PubMed [Internet]. [cited 2023 Feb 26]. Available from: https://pubmed.ncbi.nlm.nih.gov/26829826/

[28] World Health Organization. Vaccination schedule for Meningococcal disease [Internet]. [cited 2025 Mar 10]. Available from: https://immunizationdata.who.int/global/wiise-detail-page/vaccination-schedule-for-meningococcal-disease

[29] World Health Organization. Vaccination schedule for Measles [Internet]. [cited 2025 Mar 10]. Available from: https://immunizationdata.who.int/global/wiise-detail-page/vaccination-schedule-for-measles

[30] World Health Organization. Vaccination schedule for Malaria [Internet]. [cited 2025 Mar 10]. Available from: https://immunizationdata.who.int/global/wiise-detail-page/vaccination-schedule-for-malaria

[31] Thompson HA, Hogan AB, Walker PGT, Winskill P, Zongo I, Sagara I, et al. Seasonal use case for the RTS,S/AS01 malaria vaccine: a mathematical modelling study. Lancet Glob Health. 2022;10(12):e1782–92. 36400084 10.1016/S2214-109X(22)00416-8

[32] Hogan AB, Winskill P, Ghani AC. Estimated impact of RTS,S/AS01 malaria vaccine allocation strategies in sub-Saharan Africa: a modelling study. PLoS Med. 2020;17(11):e1003377. 33253211 10.1371/journal.pmed.1003377 PMC7703928

[33] Malinga J, Braunack-Mayer L, Masserey T, Cavelan A, Chandramohan D, Dicko A, et al. Performance characteristics and potential public health impact of improved pre-erythrocytic malaria vaccines targeting childhood burden. PLoS Glob Public Health. 2025;5(8):e0004549. 40758687 10.1371/journal.pgph.0004549 PMC12321141

[34] Chandramohan D, Zongo I, Sagara I, Cairns M, Yerbanga RS, Diarra M, et al. Seasonal Malaria Vaccination with or without Seasonal Malaria Chemoprevention. N Engl J Med. 2021;385(11):1005–17. 34432975 10.1056/NEJMoa2026330

[35] World malaria report. addressing inequity in the global malaria response. Geneva: World Health Organization; 2024. p. 2024.

[36] Paton RS, Kamau A, Akech S, Agweyu A, Ogero M, Mwandawiro C, et al. Malaria infection and severe disease risks in Africa. Science. 2021;373(6557):926–31. 34413238 10.1126/science.abj0089 PMC7611598

[37] World Health Organization. Establishing and strengthening immunization in the second year of life: practices for vaccination beyond infancy [Internet]. Geneva: World Health Organization; 2018 [cited 2025 Mar 7]. Available from: https://iris.who.int/handle/10665/260556

[38] De Salazar PM, Kamau A, Cavelan A, Akech S, Mpimbaza A, Snow RW, et al. Severe outcomes of malaria in children under time-varying exposure. Nat Commun. 2024;15(1):4069. 38744878 10.1038/s41467-024-48191-7 PMC11094066

[39] World Health Organization. WHO guidelines for malaria [Internet]. 2023 Aug [cited 2026 Jan 14]. Available from: https://www.who.int/publications/i/item/guidelines-for-malaria

[40] Sen S, Braunack-Mayer L, Kelly SL, Masserey T, Malinga J, Moehrle JJ, et al. Public health impact of current and proposed age-expanded perennial malaria chemoprevention: a modelling study. Sci Rep. 2025;15(1):10488. 40140443 10.1038/s41598-025-93623-z PMC11947144

[41] Runge M, Stahlfeld A, Ambrose M, Toh KB, Rahman S, Omoniwa OF, et al. Perennial malaria chemoprevention with and without malaria vaccination to reduce malaria burden in young children: a modelling analysis. Malar J. 2023;22(1):133. 37095480 10.1186/s12936-023-04564-9 PMC10124689

[42] The Impact of a combination of the RTS,S/AS01E Malaria Vaccine and Perennial Malaria Chemoprevention in Ghanaian Children [Internet]. [cited 2026 Jan 19]. (Pan African Clinical Trials Registry). Report No.: PACTR202307828402450. Available from: https://pactr.samrc.ac.za/TrialDisplay.aspx?TrialID=25559

[43] Grant J. A roadmap of priority evidence gaps for the co-implementation of malaria vaccines and perennial malaria chemoprevention. Malar J. 2025;24(1):126. 40247263 10.1186/s12936-025-05347-0 PMC12007247

[44] WHO review of malaria vaccine clinical development [Internet]. [cited 2024 Jun 27]. Available from: https://www.who.int/observatories/global-observatory-on-health-research-and-development/monitoring/who-review-of-malaria-vaccine-clinical-development

[45] Lampejo T. Monoclonal antibodies for the prevention of Plasmodium falciparum malaria: a multi-target approach? Infect Dis Lond Engl. 2023;1–5. 10.1080/23744235.2023.2274897 37921336

[46] Dv P. Editorial: The First Monoclonal Antibody Vaccine to Prevent Malaria Heralds a New Era of Malaria Vaccines to the Plasmodium falciparum Circumsporozoite Protein (PfCSP). Med Sci Monit Int Med J Exp Clin Res [Internet]. 2021 [cited 2022 Aug 25];27. 10.12659/MSM.934676 PMC8447852 34511592

[47] Schmit N, Topazian HM, Natama HM, Bellamy D, Traoré O, Somé MA, et al. The public health impact and cost-effectiveness of the R21/Matrix-M malaria vaccine: a mathematical modelling study. Lancet Infect Dis. 2024;24(5):465–75. 38342107 10.1016/S1473-3099(23)00816-2

